# Impact of DRGs-based inpatient service management on the performance of regional inpatient services in Shanghai, China: an interrupted time series study, 2013–2019

**DOI:** 10.1186/s12913-020-05790-6

**Published:** 2020-10-12

**Authors:** Lvfan Feng, Yuan Tian, Mei He, Jie Tang, Ying Peng, Chenjie Dong, Wenzhong Xu, Tao Wang, Jiangjiang He

**Affiliations:** 1 Department of Health Policy Research, Shanghai Health Development Research Center (Shanghai Medical Information Center), No.1477 Beijing (W) Road, Jing’an District, Shanghai, 200040 China; 2 Jiading Health Affair Management Center, Shanghai, China; 3 Jiading Health Commission, Shanghai, China

**Keywords:** DRGs, Performance evaluation, Service capacity, Service efficiency, Service quality, Interrupted time series

## Abstract

**Background:**

The asymmetry of information brings difficulty for government to manage public hospitals. Therefore, Jiading District of Shanghai has been establishing DRGs-based inpatient service management system (ISMS) to effectively compare the output of different hospitals through DRGs, reward desired hospital performance and enhance inpatient service capacity. However, the impact of the implementation of DRGs-based inpatient service management (ISM) policy in Jiading district is still unknow. We therefore conducted this study to evaluate the impact of DRGs-based ISM policy on the performance of inpatient service since its implementation in Jiading District, Shanghai, China in 2017.

**Methods:**

Using an interrupted time series design, we analyzed quarterly data of seven DRGs-based performance measures from the ISMS which covered all five public hospitals in Jiading District from 2013 to 2019. We utilized the segmented linear regression model to assess the change of level and trend of performance indicators before and after ISM policy. Dickey–Fuller test was used to examine the stationary of the data. Durbin-Watson test was performed to check the series autocorrelation of indicators.

**Results:**

Significant changes in the following indicators were observed since the implementation of ISM policy. The case-mix index (CMI) level decreased by 0.103 (*P* < 0.05), the trend increased by 0.008 (*P* < 0.05). The total weight level decreased by 3719.05 (*P* < 0.05), and the trend increased by 250.13 (*P* < 0.05). The time efficiency index (TEI) level increased by 0.12 (*P* < 0.05), and the trend decreased by 0.01 (*P* < 0.05). The cost efficiency index (CEI) level increased by 0.31 (P < 0.05), and the trend decreased by 0.02 (P < 0.05). No significant difference was found in the change of DRGs number, inpatient mortality of low-risk group cases (IMLRG) and inpatient mortality of medium-to-low risk group cases (IMMLRG).

**Conclusions:**

Findings highlight the role of ISM policy in improving the capacity and efficiency of regional inpatient service. Three prerequisites, including a good information system, high-quality EMR data, and a management team, are needed for other countries to implement their own ISM policy to help government manage public hospitals and improve the performance of regional inpatient service.

## Background

China is a country widely covered by social insurance and the hospital industry is dominated by state ownership and government control (public provision) with public hospital being the largest healthcare provider [1]. With significant commitment to embracing the shift to value-based healthcare, the Chinese government attaches more importance to the value of inpatient service provided by hospitals [2]. Given that capacity, efficiency and quality of services can be seen as indicators for achieving value-based healthcare [3], the improvement of hospitals in these aspects has been a focus of Chinese government [4]. However, the diversity of medical needs and the asymmetry of information in the healthcare system [5, 6] make the measurement of the output of hospitals a complex, persistent and pervasive problem. For example, considering the different types of patients admitted to hospitals, it is inappropriate to simply compare the performance of public hospitals. Therefore, it raises the issue of comparability to the traditional performance evaluation system which uses indicators such as average cost, length of stay, mortality to assess the performance of inpatient service provided by hospitals [7]. A widely used method to address this problem is to adjust the risk between cases through case-mix [8]. As early as 1852, Florence Nightingale proposed the concept of case-mix classification [9]. After that Fetter Robert developed the first generation of diagnosis related groups (DRGs) in the laboratory of Yale University in 1967 [10].

DRGs is used in medical insurance payment, budget management and performance evaluation [11, 12], because of its role in improving the transparency of hospitals and facilitating hospital performance evaluation. DRGs has achieved positive results in the United States. The growth rate of medical expenses and the length of stay were found significantly reduced in New Jersey after the application of DRGs payment system [13]. Soon after the success in the United States, many European health systems started to adopt DRGs classification system for prospective payment and health planning. In UK, France and Germany, DRGs-based payment system was shown effective in improving the efficiency, capacity and transparency of hospitals, reducing waiting time and length of stay, improving medical quality, and encouraging competition between hospitals. In Sweden and Finland, DRGs classification system was used for health planning and it has significantly improved the transparency in the planning and management of hospital services, and enhanced hospital efficiency [12].

The Chinese government introduced case-mix system into health reform in 2009 [14]. In 2010, Beijing, the capital of China, planned to adopt DRGs for medical performance management, hospital funding, and provider payment [15]. Since 2015, Jiading District of Shanghai has been establishing DRGs-based inpatient service management system (ISMS) to effectively compare the output of different hospitals through DRGs, reward desired hospital performance and enhance inpatient service capacity. However, the impact of the implementation of DRGs-based inpatient service management (ISM) policy in Jiading district is still unknow. We therefore conducted this study to evaluate the impact of DRGs-based ISM policy on the capacity, efficiency and quality of regional inpatient service in Jiading District, Shanghai. Different from previous studies which were restricted in hospital level, this study focused on the change of public hospital performance in a regional scale, which help assess the policy effects in a more comprehensive way. Moreover, this study used time series data rather than cross-sectional data as applied in many other studies, making it easier to quantify the effects of policy [12, 13, 16]. The results of this study can provide evidence and reference for other countries considering carrying out DRGs-based ISM policy.

## Methods

### Setting

Jiading District, located in the northwest of Shanghai, covers an area of 463.55 km^2^. There were 1.58 million inhabitants in Jiading District (2017). The per capita GDP in Jiading District was 20,508 USD (2017), exceeding the high-income country threshold (12,235 USD) [17]. The life expectancy in Shanghai was estimated to be 83.85 years (2017), similar to some high-income countries [18].

Several advantages in Jiading district ensure the effective operation of DRGs-based ISM in the region. First, the healthcare information system infrastructure in Jiading District is well developed and unified coding system has been implemented. Second, Jiading district has a strong staffing, equipped with a quality control team responsible for the integrity of the front page of medical records (FPMR) data and a government department specialized in healthcare service management.

There is a total of 5 public hospitals in Jiading District including Central Hospital, Nanxiang hospital, Anting Hospital, Traditional Chinese Medicine Hospital and Maternal and Child Health Care Hospital. As the largest healthcare provider, public hospitals account for appropriately 95% of outpatient and inpatient services [18].

In this study, we included all the 5 public hospitals covered in the ISMS. No private hospitals were included since they were not covered in ISMS. Therefore, the performance of regional inpatient service mentioned in this study can only represent the situation of these 5 public hospitals.

### Policy intervention

In 2015, Jiading District Health Commission released policy documents planning to introduce and use DRGs-based inpatient service management. Then Jiading District entered the stage of policy preparation, and gradually carried out the construction of electronic medical records (EMR), standardization of filling requirements for FPMR, standardization of disease classification system. Specifically, the FPMR applied the 2012 national standard version, and the coding system adopted ICD-10 and ICD-9 Shanghai version. In 2016, a district level FPMR quality control group was established to conduct supervision and training on the integrity of FPMR. In 2017, Jiading District completed the construction of DRGs-based ISMS and entered the policy implementation period.

There are five aspects for implementing DRGs-based ISM policy: (i) DRGs-based Budget: including the budget for number of cases and cost. (ii) Supervision of Inpatient Service Quality: including the quality of EMR, disease classification and performance evaluation of inpatient service based on three dimensions of capacity, efficiency and quality. (iii) Incentive Mechanism: linking the performance evaluation results with government’s investment in hospitals. (iv) Publicity of Inpatient Service Information: opening the supervision information of all DRGs to the hospital. (v) Discipline Evaluation: including the evaluation of the balanced development of different disciplines, as well as the evaluation of key disciplines of the hospital.

DRGs-based ISM policy differs from traditional approach of hospital management mainly in the following five points: (i) In the past, hospital management by government was relatively rough. The monitoring of service quantity and cost was mainly at hospital level rather than by different types of diseases. This may due to the high management cost and asymmetric of information. While through implementing ISMS, the budget management can be carried out based on DRGs, and the change in service quantity and average cost of diseases can be mastered at the disease level. (ii) In China, the health sector evaluates hospital performance on an annual basis, which induced deficiencies in three aspects compared with the performance evaluation in ISM policy. The first aspect is the data authenticity. In traditional approach of hospital management, the performance evaluation data was filled in by the hospital, rather than being retrieved through the backstage data in real time like ISMS did, which could avoid falsification of hospitals. The second aspect is the timeliness of the data. In the past, the performance of hospital was assessed once or twice a year while ISMS can achieve monthly performance evaluation, which greatly enhance the timeliness of data, and reflect the real-time performance of the hospital. The third aspect is the comparability of data. We mentioned above that traditional performance indicators such as average length of stay and average cost may have poor comparability without taking into consideration of different types of diseases. The performance indicators of ISMS can effectively solve these problems. (iii) The incentive mechanism of ISM policy can better encourage hospitals to achieve better medical output. In the past, government investment was not evidence-based. It mainly depended on the scale or losses of hospitals, instead of the output of hospitals. Through ISM, we can reward hospitals with high efficiency and productivity and maximize the use of national finance. (iv) Hospital performance can be shared with hospital in real time through ISMS. Hospitals can then obtain its own performance and compare it with other hospitals to assess whether its performance is excellent or whether there is room for improvement. (v) Finally, through the evaluation of hospital disciplines, it helps government to determine the advantages of each hospital in the region, and help the hospital to improve departments with poor performance.

### Data sources and outcome indicators

Original data came from the FPMR of all five public hospitals covered in the ISMS in Jiading District from 2013 to 2019, including more than 510,000 discharged cases and involving a total cost of 589.15 million USD. ISMS groups the cases based on diagnosis name and treatment data extracted from FPMR. In addition, the medical expenses, length of stay, mortality and other data of each case were collected to calculate performance indicators which could be reported monthly, quarterly and annually. So through the ISMS, we collected quarterly data, from 2013 to 2019, of 7 DRGs-based performance measures [19, 20] including the capacity dimension (DRGs number, case-mix index (CMI), total weight), efficiency dimension (time efficiency index (TEI), cost efficiency index (CEI)), quality dimension (inpatient mortality of low-risk group cases (IMLRG), inpatient mortality of medium-to-low risk group cases (IMMLRG)).

### Statistical analysis

2015–2016 is the preparation period of the policy, and 2017 is the actual implementation period of the policy. Therefore, this study uses the interrupted time series (ITS) design to evaluate the policy effect of DRGs-based ISM policy on the performance of regional inpatient service after its implementation in Jiading District in 2017. ITS design is considered as a strong quasi-experimental methodology in effect evaluation, which can be used to evaluate the long-term effect of a policy intervention without control group [21].

We used the segmented linear regression model to detect the change of level and trend (slope) of 7 performance measures before and after the implementation of ISM policy. The change of level indicated the change of performance measures at the time of intervention, while the change of trend indicated the long-term effect of policy. The typical segmented regression model of ITS is as follows [22, 23]:

$$ {Y}_t={\beta}_0+{\beta}_1\ast {time}_t+{\beta}_2\ast {intervention}_t+{\beta}_3\ast time\ {after\ intervention}_t+{\varepsilon}_t $$

*Y*_*t*_ stands for performance indicators, such as CMI; *time*_*t*_ is a continuous variable, indicating the time from the beginning of the observation period, taking quarter as the unit, with the value of 1, 2, 3; *intervention*_*t*_ is a binary variable, with the code of 0 before the intervention and 1 after the intervention; *time after intervention*_*t*_ is a continuous variable after the intervention, with the code of 0 before the intervention; the value after the intervention is the same as that of time. *β*_1_ represents the trend before the intervention, *β*_2_ represents the level change; *β*_3_ represents the trend change; *β*_1_ + *β*_3_ represents the trend after the intervention.

In order to verify that the data meet the requirements of ITS design, we first plotted the quarterly data of 7 performance indicators of regional DRGs-based inpatient service in Jiading District from 2013 to 2019 and visually compared the trend of each quarter of performance indicators before and after the intervention [24]. We assumed linearity of the trend lines within each segment. In addition, we used Dickey–Fuller test to examine the stationarity of time series data [25]. Durbin-Watson test was used to check the serial autocorrelation. If there is serial autocorrelation, Prais-Winsten estimation was used to correct the first-order serial correlation error [18]. All data analysis was performed using R 3.5.1.

## Results

### Overall changes in 7 regional service performance indicators in 2013–2019

**Fig. 1:**
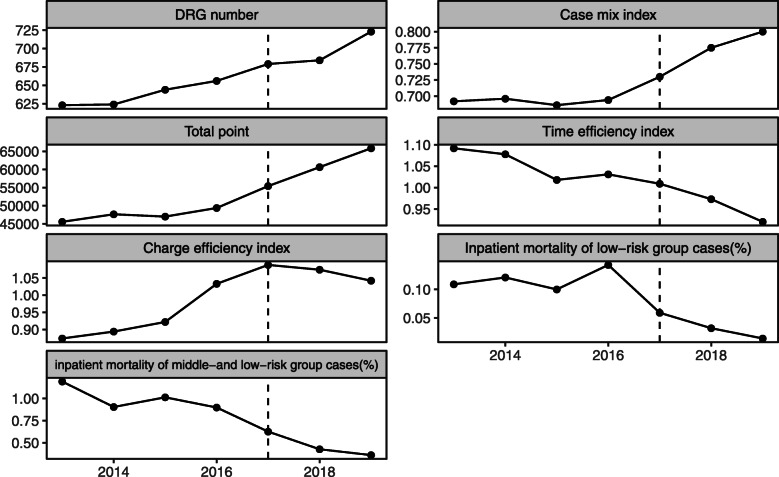
Trend of regional performance indicators in 2013–2019

Table 1 showed the annual data of 7 regional DRGs-based inpatient service performance indicators in Jiading District from 2013 to 2019. The annual growth rate was listed before and after ISM policy implemented in 2017. Figure 1 was a line chart of each annual indicator, showing the trend of each indicator.

**Table 1:** Regional medical performance indicators between 2013 and 2019

Indicator	2013	2014	2015	2016	2017	2018	2019	13–16 annual growth rate	17–19 annual growth rate
**DRGs number**	623	624	644	656	679	684	723	1.3%	2.2%
**CMI**	0.692	0.696	0.686	0.694	0.730	0.775	0.800	0.1%	3.2%
**Total weight**	45,571	47,646	47,008	49,387	55,425	60,648	65,850	2.1%	6.3%
**TEI**	1.092	1.078	1.018	1.031	1.009	0.973	0.920	−1.4%	−3.0%
**CEI**	0.874	0.894	0.922	1.033	1.088	1.074	1.042	4.6%	−1.4%
**IMLRG(%)**	0.109	0.121	0.100	0.143	0.059	0.032	0.014	7.7%	−25.7%
**IMMLRG(%)**	1.191	0.904	1.013	0.898	0.626	0.427	0.361	−6.1%	−14.1%

In capacity dimension, DRGs number, CMI and total weight all showed an upward trend. The annual growth rate of DRGs number changed from 1.3 to 2.2% before and after the policy; CMI changed from 0.1 to 3.2% before and after the policy; total weight changed from 2.1 to 6.3% before and after the policy. It was shown that the upward trend of all three indicator was accelerated after the implementation of the policy in 2017 (Table 1, Fig. 1).

In efficiency dimension, there was a downward trend in TEI and the annual growth rate decreased from - 1.4% to - 3.0% before and after the policy; the CEI started to decline after the policy with the annual growth rate changing from 4.6% to - 1.4%. Therefore, it indicated that the TEI decreased rapidly and the CEI changed from an upward trend to a downward trend after the implementation of ISM policy.

In quality dimension, the annual growth rate of IMLRG changed from 7.7% to - 25.7% before and after the policy, and the annual growth rate of IMMLRG changed from - 6.1% to - 14.1%. The downward trend of IMLRG was accelerated. The trend of IMMLRG was the same as that of the CEI, and the upward trend was changed into a downward trend.

### Results of the segmented regression analysis

**Fig. 2:**
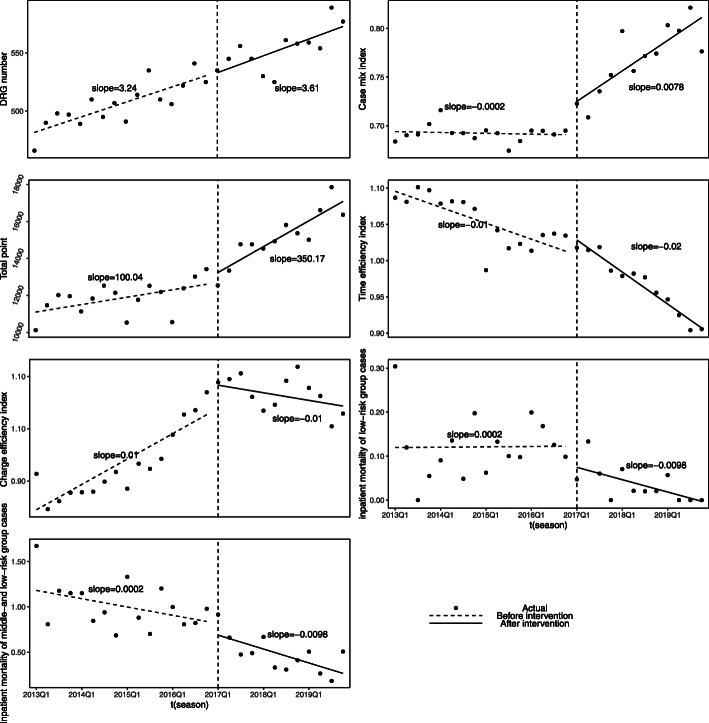
Graphic of change in performance indicators pre- and post- policy intervention

Table 2 and Fig. 2 showed the ITS analysis results of each performance indicator. In the capacity dimension, after the implementation of ISM policy, an immediate decline and an upward trend was observed in DRGs number although not statistically significant (*β*_2_ = − 6.94, *p* = 0.781; *β*_3_ = 0.37, *p* = 0.768). After the implementation of ISM policy, CMI decreased immediately. Meanwhile, the change from a slightly declining trend to a rapidly increasing trend was observed in CMI (*β*_2_ = − 0.103, *p* = 0.002; *β*_3_ =0.008, *p* < 0.001). After the implementation of ISM policy, the total weight decreased immediately and an increasing trend change was found (*β*_2_ = − 3719.05, *p* = 0.02; *β*_3_ =250.13, *p* = 0.003).

**Table 2:** Results of change in performance indicators pre- and post- policy intervention

Indicator	Variable	β	S.E.	T	P	DW	DF
**DRGs number**	Intercept	478.43	6.55	73.081	0.000***	1.930	−4.763*
	β_1_	3.24	0.68	4.792	0.000***		
	β_2_	−6.94	24.65	−0.281	0.781		
	β_3_	0.37	1.24	0.298	0.768		
**CMI**	Intercept	0.694	0.008	88.635	0.000***	1.889	−5.263*
	β_1_	−0.0002	0.001	−0.266	0.793		
	β_2_	−0.103	0.029	−3.479	0.002**		
	β_3_	0.008	0.001	5.419	0.000***		
**Total weight**	Intercept	11,000.44	395.31	27.827	0.000***	1.91	−4.844*
	β_1_	100.04	40.88	2.447	0.022*		
	β_2_	−3719.05	1488.41	−3.499	0.020*		
	β_3_	250.13	75.13	3.329	0.003**		
**TEI**	Intercept	1.10	0.01	111.191	0.000***	1.716	−5.379*
	β_1_	−0.01	0.001	−5.374	0.000***		
	β_2_	0.12	0.04	3.087	0.005**		
	β_3_	−0.01	0.002	−2.942	0.007**		
**CEI**	Intercept	0.83	0.02	35.084	0.000***	1.079***	−5.262*
	β_1_	0.01	0.002	5.090	0.000***		
	β_2_	0.31	0.09	3.665	0.001**		
	β_3_	−0.02	0.005	−3.477	0.002**		
**IMLRG**	Intercept	0.12	0.03	3.738	0.001**	1.697	−5.854*
	β_1_	0.0002	0.003	0.061	0.952		
	β_2_	0.07	0.12	0.622	0.540		
	β_3_	−0.01	0.01	−1.191	0.245		
**IMMLRG**	Intercept	1.20	0.11	10.847	0.000***	2.288	−7.127*
	β_1_	−0.02	0.01	−1.992	0.058·		
	β_2_	0.14	0.42	0.325	0.748		
	β_3_	−0.02	0.02	−0.734	0.470		

*p < 0.05; **p < 0.01; ***p < 0.001. Durbin-Watson test all indicated no autocorrelation, except CEI. Dickey–Fuller results indicated that there is no unit root and time series is stationary. The intervention study period was between 2013 to 2019, where pre-intervention was from 2013 to 2016 and post-intervention was from 2017 to 2019

In the efficiency dimension, the implementation of ISM policy was associated with a significant level increase and a declining trend change were observed in TEI (*β*_2_ =0.12, *p* = 0.005; *β*_3_ = − 0.01, *p* = 0.007). CEI also increased immediately and the trend changed from an upward trend to a downward trend, after implementing ISM policy (*β*_2_ =0.31, *p* = 0.001; *β*_3_ = − 0.02, *p* = 0.002).

In the quality dimension, after the implementation of ISM policy, the IMLRG increased immediately and the trend reversed from an upward trend to a downward trend, but there was no statistical significance (*β*_2_ =0.07, *p* = 0.54; *β*_3_ = − 0.01, *p* = 0.245). An immediate increase change and an declining trend change with no statistical significance were observed in IMMLRG after implementing ISM policy (*β*_2_ =0.14, *p* = 0.748; *β*_3_ = − 0.02, *p* = 0.47).

## Discussion

To our best knowledge, this is the first study in China using interrupted-time series design to evaluate the impact of DRGs-based ISM policy on the performance of regional inpatient services. We also firstly look at the impact of DRGs-based ISM policy in a regional scale rather than in hospital level. As shown in the results, after implementing ISM policy, the performance of regional inpatient service has changed as follows. In capacity dimension, first, the DRGs number did not change significantly, indicating that the type of diseases in Jiading District did not change much. Second, that the trend of CMI changed from decreasing to increasing indicated an increase in the regional medical resource consumption and it may due to that the complexity of cases in this region have increased. Finally, the accelerated growth of total weight showed that the increase of total inpatient service output in this region was accelerated. In efficiency dimension, the larger negative slope for TEI showed fastened decrease in the length of stay. The trend of CEI changed from upward to downward showing that the implementation of ISM policy not only controlled the increase of cost but also tended to reduce the cost. In quality dimension, despite an accelerated decline trend was found in IMLRG and IMMLRG, there was no statistical significance. This may indicate that ISM policy has not introduced significant effect on service quality by the time of evaluation.

To our best knowledge, the majority of our study results are consistent with results from previous studies in other countries. In the United States, CMI was found to be improved rapidly after the DRGs payment reform in 1980. Some studies believe that this improvement in CMI is owed not only to the implementation of DRGs-based payment system but also to the updated coding system induced by the incentive mechanism [26]. Since the results of ISMS was used as a basis for deciding government investment to hospitals rather than as a payment system in this study, accounting for about 10% of the hospital income, the impact of economic incentive should be limited. In this case, the improvement of CMI in this study may be largely due to the increased complexity of diseases, indicating that ISM policy could potentially improve the service capacity of the hospitals. According to experience from European countries, under DRGs-based payment system, hospitals have a strong incentive to increase service volumes [12]. Similarly, the accelerated growth of total weight found in this study indicates an increase in service volumes. As to efficiency dimension, the experience from the United States and European countries [12, 13] both showed that there was a significant decline in the length of stay, but the impact on medical costs were different with some studies demonstrating a decreased growth rate while others showing no effect in cost control. In our study, a significant decrease was found not only in the length of stay, but also in medical costs, indicating the important role of ISM policy in cost control. In terms of quality dimension, several studies [12, 27] have shown that DRGs had little effect on improving quality of service. Although the service quality was not improved significantly in this study, a downward trend in mortality rate was observed. If we continue to collect data, there may be significant effects in the long run.

Currently, the health care system in most countries is dominated by public hospitals, so the management of public hospitals is an issue that all governments should pay attention to. For example, consistent with our study, for the public health care systems in Finland and Sweden, the main purpose of introducing DRGs is also to measure hospital output and compare hospital performance [28]. In Finland, public hospitals receive almost all (90%) of their revenues from the municipalities, which are quite high compared with other countries with public health care systems [29]. In the past, Finnish hospitals determined their price of services based on the cost without any national guidelines and the prices varied between hospitals [30]. Therefore, the Finnish municipalities used DRGs to retrieve accurate information on cost and output of hospital operation in order to decrease cost and increase efficiency of specialized care. With a view to increasing transparency of hospital operation, the standard definitions of hospital cases in the DRGs system could allow the municipalities to compare inpatient services and their costs and prices between hospitals [29]. Our ISM policy also makes use of the effective measurement of hospital output by DRGs, and designs a perfect policy system on this basis, so as to achieve the purpose of improving inpatient service performance of public hospitals. Starting from multiple aspects, the ISM policy could improve the hospital transparency, inform government to make effective investment, enhance the hospital motivation and competition, and eventually achieve the goal of improving the performance of regional inpatient service. First, the implementation of DRGs-based ISMS, to predict the number of cases in the region and estimate the total cost, could improve hospital transparency and make it easier for government to manage public hospitals. Second, the regular supervision by quality control team on the EMR data from each hospital reduces information asymmetry. Third, the application of the evaluation and incentive mechanism by linking the results of performance evaluation to government investment to each hospital ensures the government’s full understanding of regional inpatient service performance, rewards desired hospital performance and therefore enhances hospital motivation. Moreover, it enhances the competition between hospitals by publicizing the performance of each hospital. Lastly, the data covered in ISMS helps government to better understand the development of different disciplines in each hospital therefore informs the government investment aiming to overcome the hospital shortcomings and strengthen the predominant disciplines. There are three prerequisites for implementing ISM policy including a good information system, high-quality EMR data, and a management team. With these three prerequisites, other countries could also implement their own ISM policy to help government manage public hospitals and improve the performance of regional inpatient service.

There are some limitations in this study. First, limited by time span of the data, we used quarterly data rather than annual data to ensure enough data points, but using quarter as the unit may affect the interpretation of the level change since the influence of different quarter on level change was unavoidable. As we can see from the results, the level change of most indicators was the opposite of expectation, it might be explained by the fact that there was a seasonal difference between the first quarter of 2017 and the fourth quarter of 2016. Second, as the DRGs classification system was improved and the weight was adjusted in 2019, it may have some impact on the comparability of the data of performance indicators in 2019 with the data in 2013–2018. However, considering that no major adjustment was made, the impact should be minor. Finally, as China’s health reform has lasted for 10 years [31], and a number of reforms have been launched, such as zero mark-up drug and drug pricing negotiations [18, 32]. These policies may also have some impact on the performance indicators evaluated in our study, enlarging or distorting our research results. However, China’s health reform mainly focuses on the price adjustment of drugs and medical services, with less attention on hospital management, therefore the direct impact of health reform on hospital management should be less than that in other aspects. But restricted by the limited data, we cannot find a control group which had the same type of data and had not implemented the policy. We therefore adopted a single set of ITS design with an issue that confounding due to co-interventions or other events occurring around the time of the intervention could not be completely avoided.

## Conclusion

The evidence generated by the above methods show that Jiading government has achieved the goal of effective management of public hospitals. With a good information system, high-quality EMR data, and a management team, the ISM policy played an important role in improving the capacity and efficiency of regional inpatient service. This case study provides a strong evidence and an effective example for other countries to implement their own ISM policy to help government manage public hospitals and improve the performance of regional inpatient service.

## Data Availability

The data that support the findings of this study are available from Jiading Health Affair Management Center but restrictions apply to the availability of these data, which were used under license for the current study, and so are not publicly available. Data are however available from the authors upon reasonable request and with permission of Jiading Health Affair Management Center.

## Abbreviations

DRGs: Diagnosis Related Groups
FPMR: The front pages of medical records
ITS: Interrupted time series
ISM: Inpatient service management
ISMS: Inpatient service management system
CMI: Case-mix Index
CEI: Cost Efficiency Index
TEI: Time Efficiency Index
IMLRG: Inpatient mortality of low-risk groups cases
IMMLRG: Inpatient mortality of medium-to-low risk groups cases.
